# Simple semi-high throughput determination of activity signatures of key antioxidant enzymes for physiological phenotyping

**DOI:** 10.1186/s13007-020-00583-8

**Published:** 2020-03-21

**Authors:** Lorenzo Fimognari, Rebecca Dölker, Greta Kaselyte, Camilla N. G. Jensen, Saqib S. Akhtar, Dominik K. Großkinsky, Thomas Roitsch

**Affiliations:** 1 Chr-Hansen A/S, Plant Health Innovation, Bøge Allé 10-12, 2970 Hørsholm, Denmark; 2 Department of Plant and Environmental Sciences, Section of Crop Science, Copenhagen University, Højbakkegård Allé 13, 2630 Tåstrup, Denmark; 3 Department of Plant and Environmental Sciences, Section for Transport Biology and Copenhagen Plant Science Centre, University of Copenhagen, Thorvaldsensvej 40, 1871 Frederiksberg C, Denmark; 4 Department of Adaptive Biotechnologies, Global Change Research Institute, CAS, Brno, Czech Republic

**Keywords:** Physiological phenotyping, ROS metabolism, Enzymatic assay, High throughput, Reactive oxygen species

## Abstract

**Background:**

Reactive oxygen species (ROS) such as hydrogen peroxide and superoxide anions significantly accumulate during biotic and abiotic stress and cause oxidative damage and eventually cell death. There is accumulating evidence that ROS are also involved in regulating beneficial plant–microbe interactions, signal transduction and plant growth and development. Due to the relevance of ROS throughout the life cycle and for interaction with the multifactorial environment, the physiological phenotyping of the mechanisms controlling ROS homeostasis is of general importance.

**Results:**

In this study, we have developed a robust and resource-efficient experimental platform that allows the determination of the activities of the nine key ROS scavenging enzymes from a single extraction that integrates posttranscriptional and posttranslational regulations. The assays were optimized and adapted for a semi-high throughput 96-well assay format. In a case study, we have analyzed tobacco leaves challenged by pathogen infection, drought and salt stress. The three stress factors resulted in distinct activity signatures with differential temporal dynamics.

**Conclusions:**

This experimental platform proved to be suitable to determine the antioxidant enzyme activity signature in different tissues of monocotyledonous and dicotyledonous model and crop plants. The universal enzymatic extraction procedure combined with the 96-well assay format demonstrated to be a simple, fast and semi-high throughput experimental platform for the precise and robust fingerprinting of nine key antioxidant enzymatic activities in plants.

## Background

The appearance of oxygen in the atmosphere two and a half billion years ago as a result of photosynthesis forced biological systems to adapt to this new oxidative challenge. Reactive oxygen species (ROS) produced in plant cells have taken important roles in the biology of plants, acting as indispensable signaling molecules such as in response to high light [1], regulation of cell cycle [2], seed germination [3], pathogen response [4], root development [5], etc. On the other hand, ROS can be toxic [6, 7] depending on the levels, plant developmental stage and environmental conditions. Thus, the analyses of ROS metabolism within a physiological phenotyping approach [8, 9] is gaining increasing importance for monitoring and predicting the plant performances in a multivariate natural environment. In climate change scenarios, the ability of plants to cope with quickly changing environmental conditions is key for ensuring high reproductive fitness in general and high yield of crop plants in particular.

ROS are oxygen derivatives formed during aerobic cellular metabolism in the chloroplasts, in the electron transport chain of mitochondria and in peroxisomes. It is estimated that 1% of the cellular oxygen creates ROS that when present in excess amounts lead to oxidative stress conditions and consequent damage to lipids, proteins and nucleic acids [7]. For plants, ROS are not always acting as toxic molecules, but they have been shown to play important roles in the regulation of metabolism and development. Several studies showed that ROS can alter a number of metabolic pathways during organ development and response to stresses; therefore, the precise control of ROS in terms of abundance, cellular location and time is of paramount importance. For example, specific ROS signatures have been associated with abiotic stress responses (drought, salinity, heat and light) and the ability of plants to overcome such stresses [10, 11]. Plant morphology is also affected by the metabolism of ROS, where pollen formation and root hair growth are primary examples [12, 13].

**Fig. 1:**
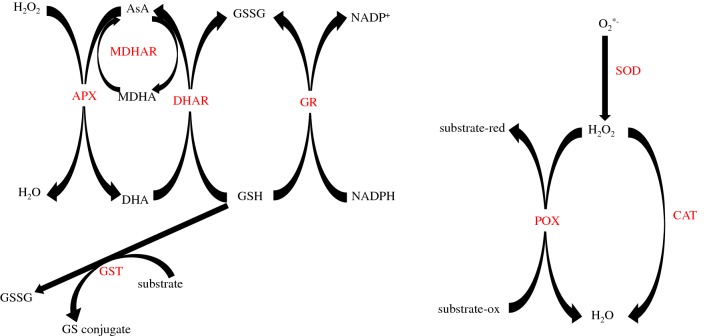
Schematic view of selected antioxidant scavenging enzymatic reactions. Ascorbic acid (AsA), monodehydroascorbate (MDHA), dehydroascorbate (DHA), oxidized glutathione (GSSG), reduced glutathione (GSH), dehydroascorbate reductase (DHAR), ascorbate peroxidase (APX), glutathione reductase (GR), monodehydroascorbate reductase (MDHAR), cytoplasmic/apoplastic peroxidases (POX, cwPOX), catalase (CAT), glutathione S-transferase (GST), superoxide dismutase (SOD)

Over-accumulation of ROS has been associated with programmed cell death whereas lower levels can stop the cell cycle and trigger secondary cell wall development [10, 11, 14–18]. An example is the overproduction of ROS under pathogen attack which is called “oxidative burst”, a key signaling event triggering the hypersensitive response, whereby the coordinated suicide of the host is executed as an attempt to limit pathogen proliferation [19]. ROS have also been shown to interplay with hormonal pathways, including ABA, ethylene and auxin, affecting plant development and the ability to alleviate biotic and abiotic stresses. Hydrogen peroxide is known to be able to activate the MAPK cascade which has implication in stress response pathways and act antagonistically in auxin transduction pathways and cell elongation [20]. ROS have also been shown to play an important role in senescence, where the peroxidation of free lipids is a key step in different senescence-inducing pathways [7]. Hence, plants employ both enzymatic and non-enzymatic antioxidant scavenging system to maintain ROS to the desired levels. ROS are found as radicals and non-radicals where the radicals forms such as superoxide radicals (O^∙–2^), perhydroxyl radical (HO^∙^_2_) and alkoxy radicals (RO) pose the biggest threat due to their greater ability to damage lipids, proteins and nucleic acids [21]. Non-radicals ROS include singlet oxygen (^1^O_2_) as well as hydrogen peroxide (H_2_O_2_) which is the most abundant ROS in plant cells. Non-enzymatic antioxidant scavenging systems include the glutathione and ascorbate pools as well as carotenoids, tocopherols and phenolics [22]. Enzymatic scavenging is achieved through the activity of different enzymes (Fig. 1): superoxide dismutase (SOD, EC:1.15.1.1), apoplastic and cytoplasmic peroxidases (cwPOX, POX, EC:1.11.1.5), catalase (CAT, EC:1.11.1.6), glutathione S-transferase (GST, EC:2.5.1.18), ascorbate peroxidase (APX, EC:1.11.1.11), monodehydroascorbate reductase (MDHAR, EC:1.6.5.4), glutathione reductase (GR, EC:1.8.1.7) and dehydroascorbate reductase (DHAR EC1:1.8.5.1). Currently, the profiling of the antioxidant enzymatic ROS scavenging fingerprint by spectrometry-based enzymatic assays is material intensive, time consuming and low-throughput. This is due to the need of unique extractions for each of the enzymatic activities to be tested, as well as the high volume of extracts and the lengthy labor time requirements for the cuvette-based assays. Therefore, typically the quantification of the level of transcripts by qPCR, microarrays or RNAseq are used as proxy to assess the activity of the encoded enzymes to draw conclusions on the underlying metabolic processes. However, these higher throughput gene expression techniques often fail to correctly reflect the resulting enzymatic activity in the plant tissue due to operational posttranscriptional and posttranslational mechanisms such as protein turnover due to stability and degradation of the enzymes, protein modifications and interactions and protein translation rates. It is estimated that in less than 50% of the cases the mRNA levels correlate with the final enzymatic activities. Thus, alternative higher throughput experimental approaches for the characterization of the plant enzymatic antioxidant defenses at the level of the enzyme activities are needed for physiological phenotyping [8] within a holistic phenomics approach [9] for basic science studies with model plants, applied, translational research with crop plants and within breeding programs.

Here, we report a semi-high throughput pipeline for the fingerprinting of nine antioxidant metabolism enzymatic activities. A single extraction developed earlier for the profiling of the carbohydrate metabolism enzymatic signatures [23] was adopted to be robust and suitable also for the analysis of antioxidant metabolism enzymatic activities. The nine previously published antioxidant enzymatic assays were miniaturized in a 96-wells plate system and the kinetic of the enzymatic reaction was followed spectrophotometrically. Different tissues were tested originating from both monocotyledonous and dicotyledonous model and crop plants. Moreover, a case study where tobacco plants have been challenged by drought and salinity, as important abiotic stresses, or pathogen infection and wounding, as relevant biotic stresses, highlighted the robustness of this screening platform as well as the ability to distinguish specific temporal dynamics of particular enzymatic activities after the stresses were imposed.

## Results

### Adaptation of the antioxidant enzymatic assays protocols to a 96-well microplate format

The previously published protocols for measuring the activity of the nine selected antioxidant enzymatic assays SOD, cwPOX, POX, CAT, GST, APX, MDHAR, GR and DHAR, [24–31] [24–30,32] [19–25,27] [17–23,25] [15–21,23] [15–21,23] [14–20,22] [13–19,21] [12–18,20] [11–17,19][10–16,18] [9–15,17] were miniaturized and optimized to a 96-well microplate format (Additional file 1: Table S1). Besides the higher throughput of the 96 well system this setup allows to conveniently follow the kinetics of each of the enzymatic reaction for quality control to ensure that the reactions are in the linear range. As the plant extract containing the activity of interest is the limiting factor in each assay, the slope of the curves reveals the enzymatic activity in the samples. This is of great advantage in comparison to the cuvette assays, which in most cases measure endpoint activities and therefore lack the inbuilt quality control. The procedure for dialysis following enzyme extraction from plant tissue was identical to a previously published report [23] and was universal for all of the antioxidant enzymatic activities tested.

**Fig. 2:**
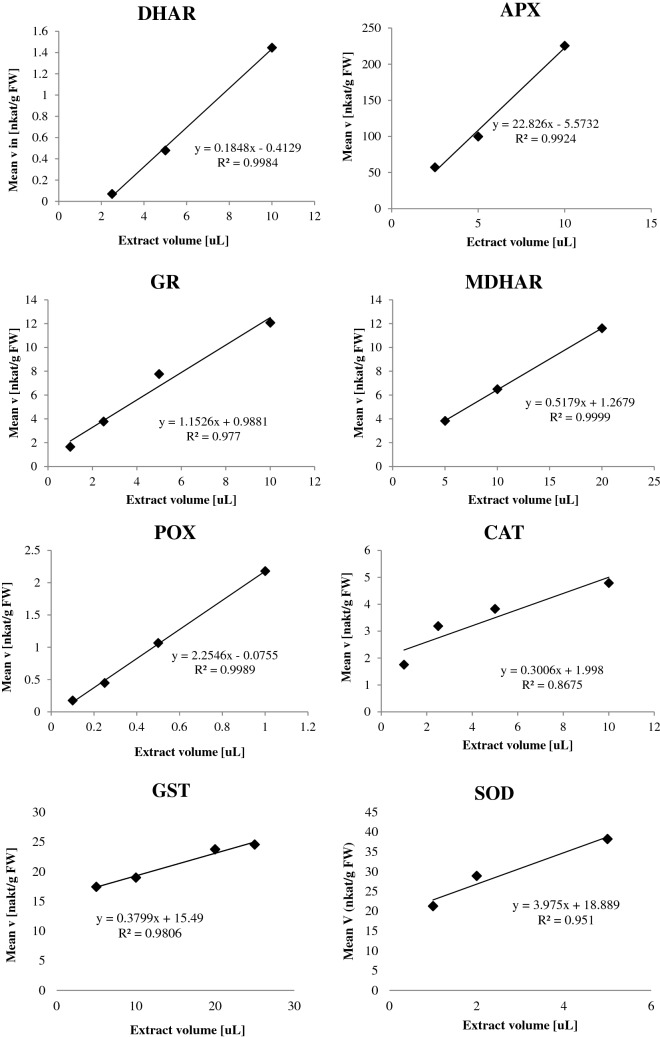
Extract dependency curves of the 9 antioxidant enzymatic activities. Dehydroascorbate reductase (DHAR), ascorbate peroxidase (APX), glutathione reductase (GR), monodehydroascorbate reductase (MDHAR), cytoplasmic/apoplastic peroxidases (POX, cwPOX), catalase (CAT), glutathione S-transferase (GST) and superoxide dismutase (SOD). Activities are normalized to the amount of fresh weight used for extraction. All the assays were performed on tobacco extracts

Extract dependency of the assays was confirmed (Fig. 2) and all assays showed a linear extract dependency response indicating that the extraction procedure and the assay setups were suitable.

We found that the adaptation of the CAT assay to a microplate format required the addition of an antifoam agent in the reaction mixture due to the formation of bubbles in the wells from the generation of O_2_ in the assay. This turned out to be an inherent problem in the 96 well microplate system where the light beam crosses the well vertically and therefore the bubbles on top of the reaction mixture can interfere with the reading, in contrast to a horizontal beam in cuvettes. The antifoam agent alleviated the problem significantly.

### Determining the antioxidant enzymatic signature in different tissues of monocotyledonous and dicotyledonous model and crop plants

The experimental platform was tested on different plant species and tissues to assess the applicability of the methodology for routine phenotyping in a wide range of plant species and tissues (Table 1, Additional file 2: Table S2). Table 1 shows the results normalized by fresh weight whereas Additional file 2: Table S2 display normalization by protein content. Leaves from the model plant *Arabidopsis thaliana* and the crops maize and barley were used for this purpose. Additionally, root samples of maize and sugar beet were tested as well. As expected, the different samples revealed an unique activity signature for the nine different enzymes. For example, APX activity is relatively low in *Arabidopsis* leaves and higher in the other samples tested. Conversely, *Arabidopsis* leaves have the highest GR activity. These results demonstrate the potential and robustness of the system in detecting alteration of the antioxidant enzymatic profile in different plant material.

**Table 1:** Activity profile of the nine antioxidant enzymatic signature in different plants and tissue

	Arabidopsis leaves	Maize leaves	Maize roots	Sugar beet roots	Barley leaves
SOD	44.2 ± 2.05	15.4 ± 5.50	26.4 ± 5.34	8.02 ± 4.08	21.1 ± 1.05
POX	0.431 ± 0.106	3.71 ± 1.07	N.D.	0.131 ± 0.0220	8.81 ± 0.926
cwPOX	8.18 ± 1.01	1.20 ± 0.156	1.06 ± 0.931	1.27 ± 0.0373	4.73 ± 0.283
CAT	2.17 ± 0.300	0.226 ± 0.0581	0.0389 ± 0.0455	0.217 ± 0.0470	0.761 ± 0.123
APX	N.D.	2.73 ± 1.62	3.23 ± 4.04	N.D.	1.97 ± 0.977
MDHAR	3.02 ± 0.951	N.D.	N.D.	0.464 ± 0.0402	0.302 ± 0.0633
DHAR	5.02 ± 0.485	N.D.	N.D.	0.353 ± 0.103	0.235 ± 0.0238
GR	6.91 ± 0.514	1.45 ± 0.499	0.0199 ± 0.0348	N.D.	0.495 ± 0.203
GST	8.25 ± 0.0597	0.0298 ± 0.0126	0.183 ± 0.0539	4.28 ± 0.122	5.11 ± 0.0935

The values are normalized per fresh weight (Mean v [nkat/g FW]) ± standard deviation

The activities for Arabidopsis leaves, maize leaves and maize roots are average of 3 independent plant replicates whereas for sugar beet roots and barley leaves 3 distinct extractions were performed on the same plant sample, consisting of at least 15 individual plants for barley leaves and 3 plants for sugar beet roots

*N.D.* not detectable

### Case study: fingerprinting the impact of biotic and abiotic stresses on the activity signatures of antioxidant metabolism enzymes in tobacco leaves

ROS have been previously reported to accumulate in plants undergoing abiotic and biotic stresses [2]. In order to assess the potential of this experimental pipeline in profiling the plant antioxidant enzymatic response to plant age and both biotic and abiotic stress, three experiments were designed. Greenhouse-grown *Nicotiana tabacum* was exposed to drought stress, salt stress, and pathogen infection and wounding at 60 days after sowing and the temporal dynamics of the various antioxidant enzymatic activities were measured.

**Fig. 3:**
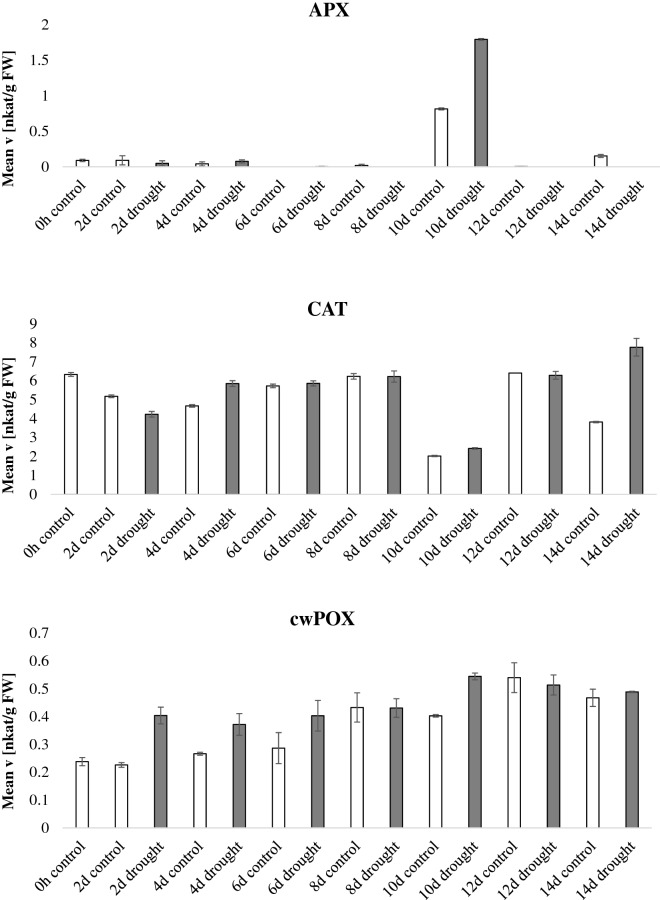

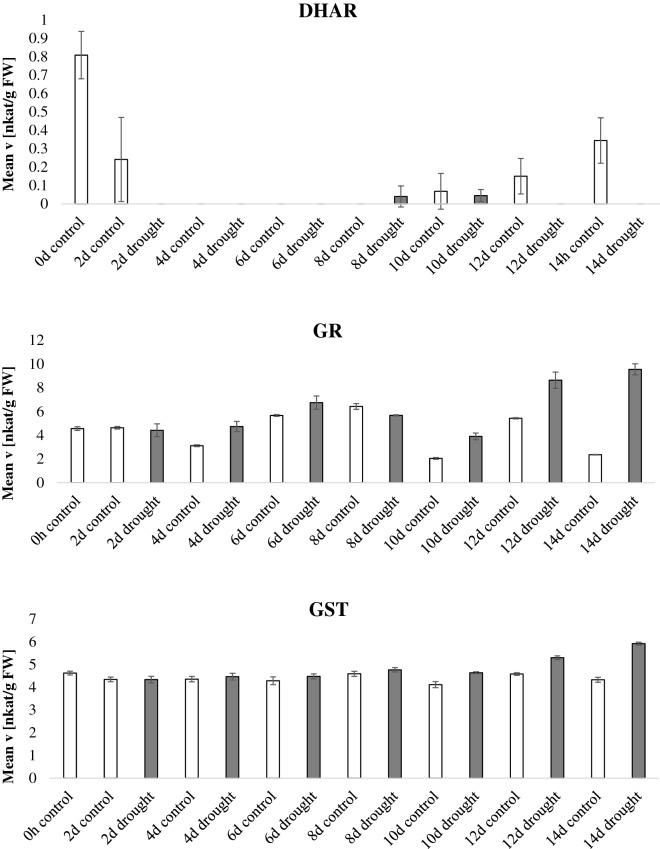

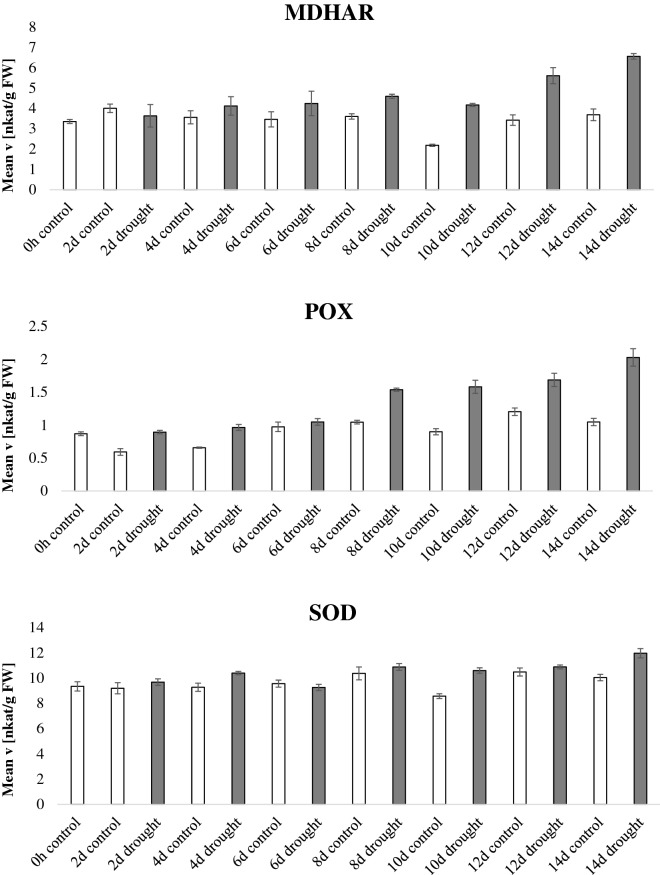
Antioxidant enzymatic activities during drought. *Nicotiana tabacum* plants were grown for 60 days in the greenhouse, thereafter, water was withheld for half of the plants whereas the other half was kept watered. Leaves were harvested every 2 days for a total of 14 days after watering was stopped and the enzymatic activities of the 9 antioxidant scavenging enzymes were tested and normalized by fresh weight. Bars indicate standard deviations of three independent biological replicates

In the drought experiment (Fig. 3, Additional file 3: Figure S1), watering was completely withheld starting at day 60 after sowing and samples were harvested every 2 days for 14 days. Figure 3 expresses the data normalized by fresh weight whereas Additional file 3: Figure S1 displays normalization by protein content; notably the enzymatic activities show the same trends regardless the normalization method. GR and POX activity increased during the drought period. CAT activity was higher in the last day of the drought stress (14 days of drought) and DHAR activity was inversely correlated to drought stress. Similar data were presented in previous reports [32–34]. The other enzymatic activities did not display a consistent activity pattern related to plant age or stress condition. It is to notice however a particular deviation of the activities of the samples harvested at day 10, especially for APX and CAT activities, compared to the preceding and following time points. The APX activity was increased more than tenfold at day 10, whereas the CAT activity was reduced. A possible explanation for this trend is that at the time of harvest (day 10) the plants were experiencing an additional stress related to varying greenhouse conditions. Nevertheless, this “outlier” data point proves further the robustness and specificity of this experimental pipeline in detecting enzymatic signature which reflect specific plant physiological states. Thus, this method can be used to eventually identify the impact of unwanted external factors within an experiment.

**Fig. 4:**
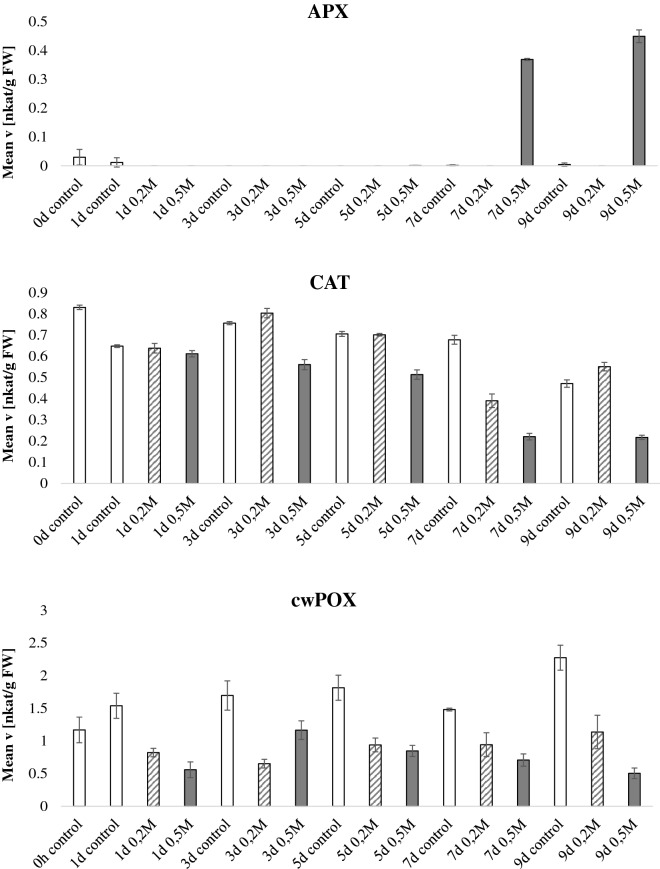

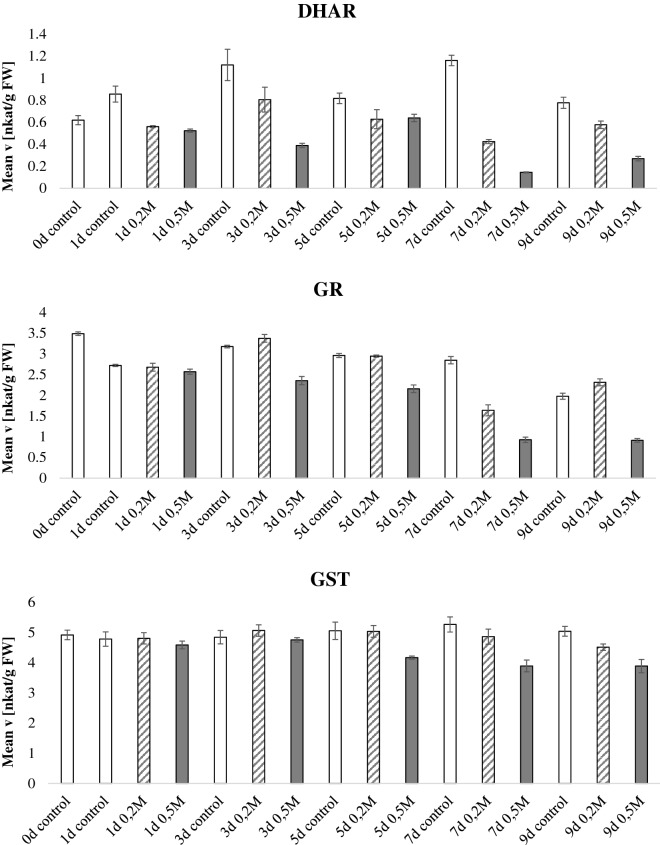

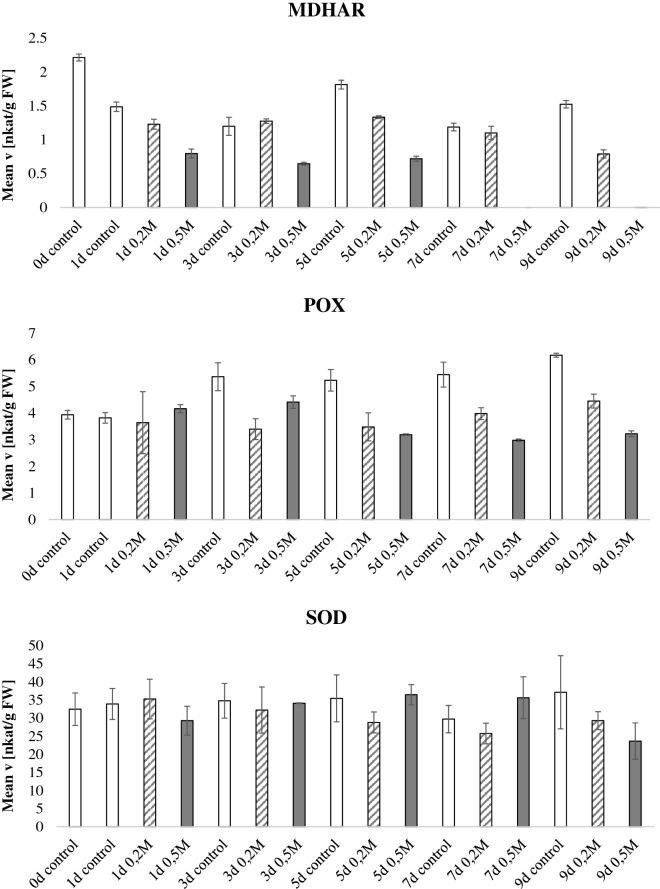
Antioxidant enzymatic activities during salt stress. *Nicotiana tabacum* plants were grown for 60 days in the greenhouse, thereafter, detached leaves were fed with 0.2 M NaCl or 0.5 M NaCl via their petioles. Leaves were sampled every 2 days for a total of 14 days after NaCl application and the enzymatic activities of the 9 antioxidant scavenging enzymes were tested and normalized by fresh weight. Bars indicate standard deviations of three independent biological replicates

In the salt stress experiment (Fig. 4, Additional file 4: Figure S2), detached leaves were fed with the indicated two concentrations of NaCl via their petioles. Figure 4 expresses the data normalized by fresh weight whereas Additional file 4: Figure S2 display normalization by protein content. Unlike the drought experiment, there were differences between the fresh weight and protein normalized data. There was a pattern for higher activities in the protein normalized data at later stage of salt stress, especially for GST, SOD, POX at day 7 and 9 in the 0.5 M NaCl treatment. This was due to the fact that the protein content of these salt stresses samples was particularly low meaning that although the specific absolute enzymatic activity in the leaf was not particularly high, the proportion of these enzymatic activities as compared to the whole protein pool was high. APX had a 100 fold increase in activity at the latest time point recorded (day 7 and 9) in the 0.5 M NaCl treatment but not in 0.2 M NaCl or in control. Higher activity of APX in plants exposed to high salinity was previously reported [35]. MDHAR and cwPOX decreased at later stages of the 0.2 and 0.5 M NaCl treatment. The other enzymatic activities did not display a consistent activity pattern related to plant age or stress condition.

**Fig. 5:**
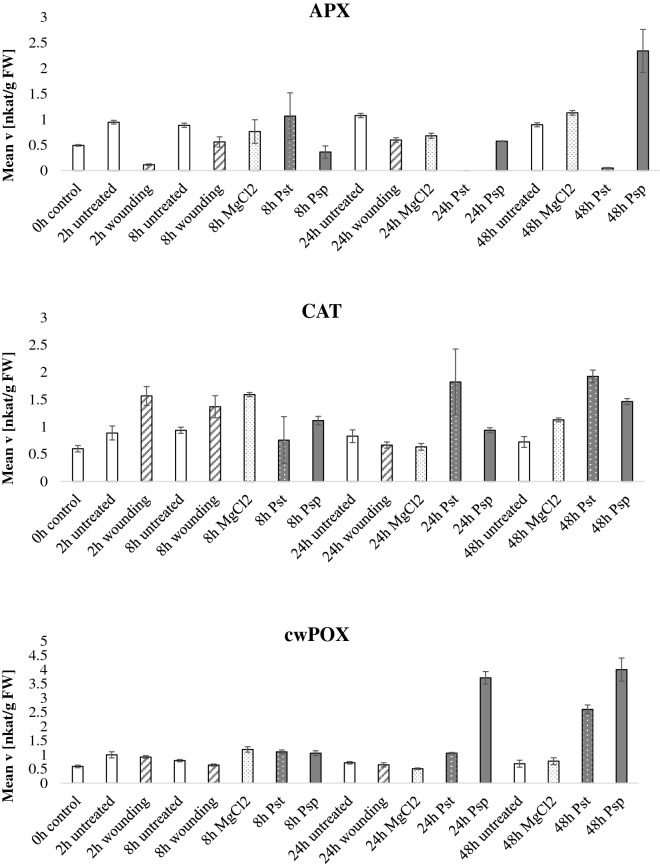

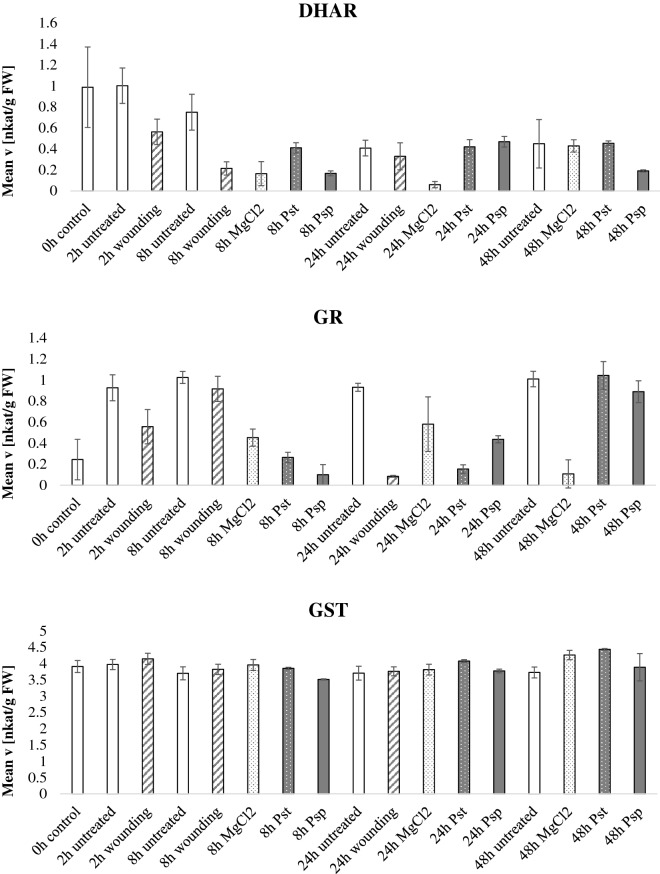

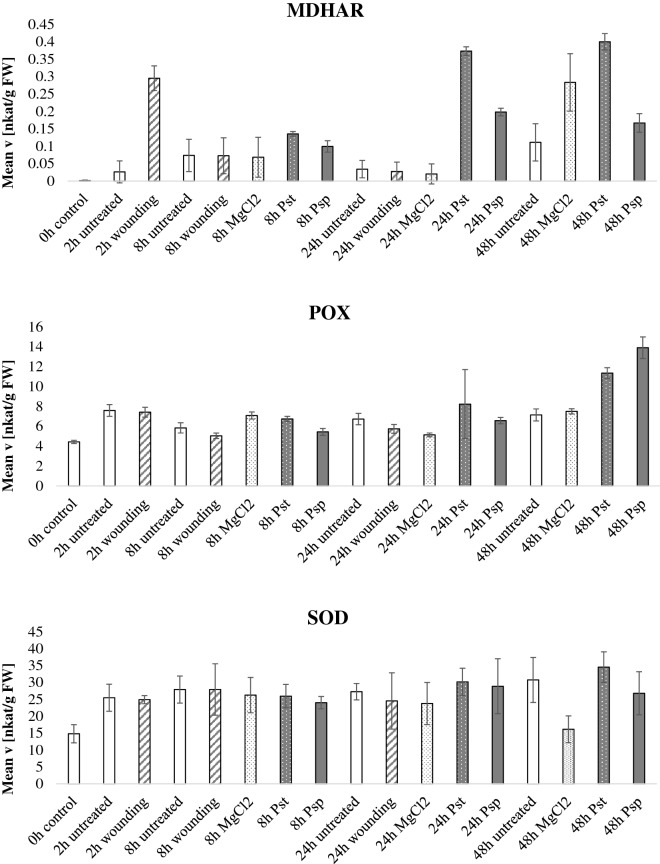
Antioxidant enzymatic activities during pathogen stress. *Nicotiana tabacum* plants were grown for 60 days in the greenhouse, thereafter, the plants were treated with either the plant pathogen *Pseudomonas syringae* pv tabaci (Pst) which is virulent in *Nicotiana tabacum*, the avirulent strain *Pseudomonas syringae* pv phaseolicola (Psp), mechanical wounding, MgCl_2_ as a control or untreated plants. Leaves were harvested at 2, 8, 24 and 48 h after treatment and the enzymatic activities of the ten antioxidant scavenging enzymes were tested and normalized by fresh weight. Bars indicate standard deviations of three independent biological replicates

In the pathogen infection experiment (Fig. 5, Additional file 5: Figure S3), the hemibiotrophic model pathogen *Pseudomonas syringae* pv. *tabaci* (Pst) was inoculated in the abaxial side of the leaves. Figure 5 expresses the data normalized by fresh weight whereas Additional file 5: Figure S3 display normalization by protein content and like in the drought experiment the enzymatic activities show the same trends regardless the normalization method. Mock treatment consisted of MgCl_2_ inoculation. Wounding treatment was included as mechanical damage and untreated plants were used as a control. Moreover, the treatment with the avirulent *P. syringae* pv. *phaseolicola* (Psp) was included in the analysis as it was previously shown that there can be differences in the antioxidant defenses elicited by virulent and avirulent strains [36]. APX activity decreased at 24 h and 48 h post Pst infection whereas the enzymatic activity at 48 h after Psp treatment increased as compared to control. It has previously been reported that pathogenic bacteria can produce exopolysaccharides which in turn alter ascorbate metabolism in plants [37]. CAT, GST, SOD, DHAR and GR did not show any specific pattern. MDHAR activity spiked 2 h after wounding and at late stages of Pst interaction. Positive correlation between MDHAR mRNA transcripts and wounding was reported in a previous study [38] as well as higher MDHAR activities post pathogen infection [39]. POX activities increased at 48 h after Pst and Psp inoculation, POX activity was previously observed to be increased at later stages of the plant–pathogen interaction [40]. cwPOX were also high in the bacterial inoculation, especially for 48 h post inoculation. Consistently, increased apoplastic cwPOX activity after *Pseudomonas syringae* infection was previously reported [41].

## Discussion

The protocol established in this study to determine an antioxidant activity signature complements the previously established protocols for enzymatic profiling of carbohydrate metabolism [23, 42–44, 51] that proved to be very useful in various studies [45–47]. Notably, the extraction protocol described in this study for the nine antioxidant activities is compatible with the experimental platform described earlier by us to determine 13 key enzymes of carbohydrate metabolism [23], that had been extended to a fructan active enzyme [51]. Thus, with a single extraction both primary carbohydrate metabolism and antioxidant metabolism can be probed from the same sample at the level of the enzymatic activities based on measurements of a single extract, allowing direct comparisons.

This methodology offers clear advantages over the standard cuvette assays because the miniaturization in 96-well microplate system reduces the amount of extract required for the reaction and decreases both labor time and consumables costs. Moreover, this setup allowed the convenient monitoring of the kinetic of each assay, permitting the determination of linear range of the enzyme reaction. This feature provides an in-built quality control which significantly increases the robustness of the assays. It is immediately possible to identify whether the reactions are in a linear range or whether adjustments of the extract volume are eventually required to avoid over or under estimations of the activities. Furthermore, the possibility to discriminate between the intracellular and cell wall bound enzymatic activities by processing the plant material with extraction buffer containing different salt concentrations adds on the understanding on the subcellular compartmentalization in the context of antioxidant defenses.

With only few exceptions, we could fingerprint the nine enzymatic activities of samples representing different tissues and plant species, highlighting the wide range of detection of the assays. It remains to be determined whether the extraction procedure needs to be adapted for phenol rich tissue such as grapevine or gymnosperms, as it has been the case of carbohydrate metabolism enzymes [52]. The plant responses to the oxidative stress imposed by challenging environmental conditions caused by biotic and abiotic stresses have the potential to become an important phenotypic trait to select for next generation crops. Within climate change scenarios the ability of plants to buffer sudden oxidative responses may be a desirable trait for plant breeding and it could be used as a monitoring tool for semi-high throughput screening of plant fitness. We conducted a case study in which *Nicotiana tabacum* was exposed to drought, salinity, pathogen and wounding stress. Remarkably, the fingerprint signature of the nine different enzymes showed distinct differences both with respect to the individual activities as well as their temporal dynamics in response to the different stresses imposed, thus providing knowledge not only on the stress levels of the plant but also on the specific type of stress. This information complements other destructive and non-destructive phenotyping techniques to assess the alteration in biochemical homeostasis caused by external environmental stimuli.

The established analytical platform to analyze the activities by a semi-high throughput technique will complement the analyses of mRNA levels by qPCR or truly high-throughput techniques. Thus, it will be possible to overcome the limitation of often poor correlation between transcript levels and the enzymatic activity in the tissue that ultimately determines the phenotype [53]. Furthermore, it will be possible to assess the possible involvement of epigenetic, posttranscriptional and posttranslational mechanisms [8, 9]. Since most of the antioxidant metabolism are conserved in many organisms it remains to be determined whether the established procedure can also be applied to other eukaryotes or prokaryotes, as it has been successfully possible for several enzyme of carbohydrate metabolism [53]. Wet chemistry based physiological analytical methods are inherently limited in throughput and thus typically are not considered for practical applications. The established protocol not only targets key activities for resource allocation but also abiotic and biotic stress responses which are highly relevant for the mechanistic determination of stress resiliency within climate change scenarios. Thus, this analysis platform will not only be relevant for basic research but also allows the integration of their results in pre-breeding and breeding programs.

## Conclusion

This method allows a reliable, cost and time effective semi-high throughput fingerprinting of the antioxidant enzymatic activities in model and crop plant tissues experiencing very different stress conditions. The extraction procedure is simple and universal for all enzymatic activities which derives from a previous work in our lab aimed at fingerprinting 13 key carbohydrate metabolism enzymes [23]. The assays are cost efficient and can be performed in any lab with access to a plate reader and thus do not require investment for expensive instrumentation. This analytical platform can be used for routine assessment of antioxidant metabolism of plants from controlled environment studies as well as from field experiments. The knowledge generated by these experiments will allow moving one step further to the monitoring and understanding how the interaction between genes and the environment shape the complex molecular and physiological events determining plant yield and fitness. We envision that the use of this methodology will help understanding enzymatic plasticity of plants undergoing multiple stresses, for example in field conditions and in environmental change scenarios. Moreover, it will increase the understanding the mechanisms behind stress tolerance of plants treated with beneficial microorganisms. Thus, this new experimental platform should be of particular relevance to implement a truly physiological phenotyping at the level of enzyme activity signatures into a holistic, multidimensional functional phenomics approach [8, 9].

## Methods

### Plant material handling and inoculation conditions

*Arabidopsis thaliana* (Col-0) was sown in pots containing pith soil and exposed to stratification at 4 °C in the darkness for 48–72 h. Afterwards they were placed in growth chambers with a light cycle of 16 h light (fluorescent light tubes with 130 µmol/m^2^/sec light intensity) and 8 h darkness and a day/night temperature of 22/18 °C. Rosettes of 4 weeks old plants were harvested, snap frozen in liquid nitrogen and stored at − 80 °C. Barley, maize and tobacco were grown in the greenhouse provide a daily light cycle of 16 h light with supplemented artificial light. Harvest was performed by snap freezing in liquid nitrogen and storage at − 80 °C. Barley and maize were grown in greenhouse at the same day/night light cycle and were harvested at 14 and 37 days post sowing respectively.

For the *Nicotiana tabacum* cv. Petit Havana SR1 drought stress case study, water was withheld at day 60 after sowing. For the salt stress case study, detached leaves from 60 days old plants were fed with 0.2 or 0.5 M NaCl via their petioles. For the pathogen stress case study, *Pseudomonas syringae* was pre-grown overnight at 28 °C. The day of the inoculation, fresh media was added to the culture flask and the OD600 was monitored until it reached OD_600_ = 0.02. Cultures were spun in a centrifuge, the supernatant was discarded and the pellet was resuspended in the same volume of 10 mM MgCl_2_. Leaves of 60 days old *N. tabacum* plants were inoculated by syringe infiltration on the abaxial side of the leaves. 10 mM MgCl_2_ infiltration was used as a mock as well as mechanically wounded leaves with razor blades [48].

### Protein/enzyme extraction

Enzyme extraction was performed as it was previously described [8]. Plant material was ground with a mortar and pestle in liquid nitrogen. The ground material was collected and approximately 0.5 g of plant material was weighted accurately in a 2 mL tube and a tip of spatula (1–3 mg) of polyvinylpolypyrrolidone was added to each tube. 1 mL of extraction buffer was added to each tube (40 mM Tris HCl pH7.6, 1 mM EDTA, 0.1 mM phenylmethane sulfonyl fluoride, 1 mM Benzamidine, 24 µM NADP, 14 mM β-Mercaptoethanol) and the samples were mixed in a rotary shaker for 30 min at 4 °C. The samples were then centrifuged for 10 min at 4 °C at 12,000 rpm. The supernatant containing the intracellular enzymes was dialyzed overnight against 20 mM phosphate buffer pH7.6 using dialysis tubing. For extraction of cwPOX activity present in the apoplast, the pellet from the previous centrifugation step was washed three times in MilliQ water and then 1 mL of high salt buffer (40 mM Tris HCl pH7.6, 15 mM EDTA, 3 mM MgCl_2_, 1 M NaCl) was added to the tubes and the samples were mixed in a rotary shaker at 4 °C overnight. The samples were then centrifuged for 10 min at 4 °C at 12,000 rpm and the supernatant was dialyzed overnight against 20 mM phosphate buffer pH7.4. Intracellular and cell wall enzymatic fractions were stored at − 20 °C until use.

### Kinetic assays procedures

All kinetic assays were performed in a 96-well plate setup (Greiner Bio One, Kremsmünster, Austria) in a Biotex synergy HTX plate reader, using variable extract volume (0.5–25 µL) and a total assay volume of 160 µL in each well. Enzymatic activities were normalized by fresh weight (main text) or protein content (Additional files 3, 4, 5) [49].

APX (ascorbate peroxidase) activity was measured with a modified procedure from [30]. In each well the extract was mixed with 50 mM K_2_HPO_4_/KH_2_PO_4_ buffer pH7.6; 0.25 mM ascorbate and 0.5 mM H_2_O_2_. Absorbance was recorded at 290 nm, 30 °C for 40 min and the slope of the curve representing the oxidation of ascorbate was recorded. For control reactions H_2_O_2_ was omitted. To quantify the APX enzymatic activity the extinction coefficient 2.8 [mM^−1^*cm^−1^] was used.

CAT (catalase) activity was measured with a modified procedure from [31]. In each well the extract was mixed with 50 mM K_2_HPO_4_/KH_2_PO_4_ buffer pH7, 0,001% Antifoam 204 and 100 mM H_2_O_2_. Absorbance was recorded at 240 nm, 30 °C for 40 min and the slope of the curve representing the disappearance of H_2_O_2_ was recorded. For control reactions H_2_O_2_ was omitted. To quantify the CAT enzymatic activity the extinction coefficient 43.6 [mM^−1^*cm^−1^] was used.

DHAR (dehydroascorbate reductase) activity was measured with a modified procedure from [26]. In each well the extract was mixed with 100 mM K_2_HPO_4_/KH_2_PO_4_ buffer pH6.5, 5 mM reduced glutathione (GSH) and 0.2 mM dehydroascorbic acid (DHA). Absorbance was recorded at 265 nm, 25 °C for 40 min and the slope of the curve representing the formation of ascorbate was recorded. For control reactions DHA was omitted. The enzymatic activities were further corrected by subtracting the non-enzymatic formation of ascorbate by including in the 96-well plate a column which did not have any extract added. To quantify the DHAR enzymatic activity the extinction coefficient 2.8 [mM^−1^*cm^−1^] was used.

GR (glutathione reductase) activity was measured with a modified procedure from [27]. In each well the extract was mixed with 100 mM Tris HCl pH7.8, 0.2 mM NADPH and 0.6 mM oxidized glutathione (GSSG). Absorbance was recorded at 340 nm, 30 °C for 40 min and the slope of the curve representing the disappearance of NADPH was recorded. For control reactions GSSH was omitted. To quantify the GR enzymatic activity the extinction coefficient 6.22 [mM^−1^*cm^−1^] was used.

GST (glutathione S-transferase) activity was measured with a modified procedure from [29]. In each well the extract was mixed with 100 mM K_2_HPO_4_/KH_2_PO_4_ buffer pH7.4, 1 mM 2,4-Dinitrochlorobenzene (CDNB) and 1 mM reduced glutathione (GSH). Absorbance was recorded at 334 nm, 30 °C for 40 min and the slope of the curve representing the formation of (2,4-dinitrophenyl)glutathione (DNP-GS) was recorded. For control reactions CDNB was omitted. To quantify the GST enzymatic activity the extinction coefficient 9.6 [mM^−1^*cm^−1^] was used.

MDHAR (monodehydroascorbate reductase) activity was measured with a modified procedure from [25, 50]. In each well the extract was mixed with 50 mM K_2_HPO_4_/KH_2_PO_4_ buffer pH7.2, 0.25 mM NADH, 1U/mL Ascorbic acid oxidase and 2 mM ascorbate. Absorbance was recorded at 340 nm, 30 °C for 40 min and the slope of the curve representing the disappearance of NADPH was recorded. For control reactions ascorbate was omitted. To quantify the MHDAR enzymatic activity the extinction coefficient 6.22 [mM^−1^*cm^−1^] was used.

POX (peroxidase) and cwPOX (cell wall bound peroxidase) activity was measured with a modified procedure from [28]. In each well the extract was mixed with 100 mM K_2_HPO_4_/KH_2_PO_4_ buffer pH7.2 mM guaiacol and 0.15 mM H_2_O_2_. Absorbance was recorded at 450 nm, 30 °C for 40 min and the slope of the curve representing the formation of tetraguaiacol was recorded. For control reactions H_2_O_2_ was omitted. To quantify the POX enzymatic activity the extinction coefficient 25.5 [mM^−1^*cm^−1^] was used.

SOD (superoxide dismutase) activity was measured with a modified procedure from [24]. In each well the extract was mixed with 50 mM K_2_HPO_4_/KH_2_PO_4_ buffer pH7.8, 0.1 mM EDTA, 0.05 mM cytochrome c, 10 mM xanthine and 0.0002U/mg xanthine oxidase. Absorbance was recorded at 550 nm, 25 °C for 40 min and the slope of the curve representing the inhibition of the oxidation of cytochrome c was recorded. For control reactions xanthine or the extract was omitted.

## Supplementary information

**Additional file 1: Table S1.** Experimental setup of the chemicals used in each enzymatic assay. In cases where the extract volume added was smaller than 25μL, buffer was added to reach a total extraction volume of 25μL in each well. In every well, the total volume of the reaction was 160 μL.

**Additional file 2: Table S2.** Activity profile of the nine antioxidant enzymatic signature in different plants and tissue. The values are normalized per mg protein (Mean v [nkat/mg prot]) ± standard deviation; N.D. not detectable. The activities for Arabidopsis leaves, maize leaves and maize roots are average of 3 independent plant replicates whereas for sugar beet roots and barley leaves 3 distinct extractions were performed on the same plant sample, consisting of at least 15 individual plants for barley leaves and 3 plants for sugar beet roots.

**Additional file 3: Figure S1.** Antioxidant enzymatic activities during drought. Nicotiana tabacum plants were grown for 60 days in the greenhouse, thereafter, water was withheld for half of the plants whereas the other half was kept watered. Leaves were harvested every two days for a total of 14 days after watering was stopped and the enzymatic activities of the 9 antioxidant scavenging enzymes were tested and normalized by protein content. Bars indicate standard deviations of three independent biological replicates.

**Additional file 4: Figure S2.** Antioxidant enzymatic activities during salt stress. Nicotiana tabacum plants were grown for 60 days in the greenhouse, thereafter, detached leaves were fed with 0,2M NaCl or 0,5M NaCl via their petioles. Leaves were sampled every two days for a total of 14 days after NaCl application and the enzymatic activities of the 9 antioxidant scavenging enzymes were tested and normalized by protein content. Bars indicate standard deviations of three independent biological replicates.

**Additional file 5: Figure S3.** Antioxidant enzymatic activities during pathogen stress. Nicotiana tabacum plants were grown for 60 days in the greenhouse, thereafter, the plants were treated with either the plant pathogen Pseudomonas syringae pv tabaci (Pst) which is virulent in Nicotiana tabacum, the avirulent strain Pseudomonas syringae pv phaseolicola (Psp), mechanical wounding, MgCl2 as a control or untreated plants. Leaves were harvested at 2, 8, 24 and 48 hours after treatment and the enzymatic activities of the ten antioxidant scavenging enzymes were tested and normalized by protein content. Bars indicate standard deviations of three independent biological replicates.

## Data Availability

All data generated or analysed during this study are included in this published article and Additional files 1, 2, 3, 4, 5.

## Abbreviations

ABA: Abscisic acid
APX: Ascorbate peroxidase
CAT: Catalase
cwPOX: Apoplastic peroxidases
DHAR: Dehydroascorbate reductase
GR: Glutathione reductase
GST: Glutathione S-transferase
MAPK: Mitogen activated protein kinase
MDHAR: Monodehydroascorbate reductase
POX: Cytoplasmic peroxidases
Psp: *Pseudomonas syringae* pv. *phaseolicola*
Pst: *Pseudomonas syringae* pv. *tabaci*
ROS: Reactive oxygen species
SOD: Superoxide dismutase
