# Supplementary material for: Simple semi-high throughput determination of activity signatures of key antioxidant enzymes for physiological phenotyping
Source: Plant Methods. 2020 Mar 21;16:42. doi: 10.1186/s13007-020-00583-8 (PMC7085164; doi:10.1186/s13007-020-00583-8)
Supplement: Supplementary file 1 — Additional file 1: Table S1. Experimental setup of the chemicals used in each enzymatic assay. In cases where the extract volume added was smaller than 25μL, buffer was added to reach a total extraction volume of 25μL in each well. In every well, the total volume of the reaction was 160 μL. [file 13007_2020_583_MOESM1_ESM.docx]

|  | **Concentration in each well** | **Typical extract volume (uL)** |
| --- | --- | --- |
| **APX** | 50mM KPO4 Buffer pH 7.6 + 0,25mM Ascorbate + 0,5mM H_2_O_2_ | 5-25 |
| **CAT** | 50mM KPO4 Buffer pH 7 + 0,001% Antifoam + 100mM H2O2 | 5-25 |
| **DHAR** | 100mM KPO4 Buffer pH 6,5 + 5mM Glutathione reduced (GSH) + 0,2mM Dehydroascorbic acid (DHA) | 5-25 |
| **GR** | 100mM Tris HCl pH 7.8 + 0,2mM NADPH + 0,6mM Glutathione oxidized (GSSG) | 5-25 |
| **GST** | 100mM KPO4 Buffer pH 7,4 + 1mM Glutathione reduced (GSH) + 1mM 2,4-Dinitrochlorobenzene (CDNB) | 5-25 |
| **MDHAR** | 50mM KPO4 Buffer pH 7,2 + 0,25mM NADH + 1U/mL Ascorbic acid Oxidase (AAO) + 2mM Ascorbate | 5-25 |
| **POX, cwPOX** | 100mM KPO4 Buffer pH 7 + 2mM Guaiacol + 0,15mM H2O2 | 1-10 |
| **SOD** | 50mM KPO4 Buffer pH 7,8 + 0,1mM EDTA + 0,05mM Cyt c + 10mM Xantine | 1-10 |

Table S1: **Experimental setup of the chemicals used in each enzymatic assay**. In cases where the extract volume added was smaller than 25uL, buffer was added to reach a total extraction volume of 25uL in each well. In every well, the total volume of the reaction was 160uL.
