# Supplementary material for: Simple semi-high throughput determination of activity signatures of key antioxidant enzymes for physiological phenotyping
Source: Plant Methods. 2020 Mar 21;16:42. doi: 10.1186/s13007-020-00583-8 (PMC7085164; doi:10.1186/s13007-020-00583-8)
Supplement: Supplementary file 2 — Additional file 2: Table S2. Activity profile of the nine antioxidant enzymatic signature in different plants and tissue. The values are normalized per mg protein (Mean v [nkat/mg prot]) ± standard deviation; N.D. not detectable. The activities for Arabidopsis leaves, maize leaves and maize roots are average of 3 independent plant replicates whereas for sugar beet roots and barley leaves 3 distinct extractions were performed on the same plant sample, consisting of at least 15 individual plants for barley leaves and 3 plants for sugar beet roots. [file 13007_2020_583_MOESM2_ESM.docx]

|  | **Arabidopsis leaves** | **Maize leaves** | **Maize roots** | **Sugar beet roots** | **Barley leaves** |
| --- | --- | --- | --- | --- | --- |
| **SOD** | 2,37 +/- 0,0496 | 0,128 +/- 0,0449 | 0,156 +/- 0,0319 | 0,643 +/- 0,327 | 0,801 +/- 0,0628 |
| **POX** | 0,104 +/- 0,0250 | 1,09 +/- 0,243 | N.D. | 0,211 +/- 0,0354 | 6,71 +/- 0,992 |
| **cwPOX** | 6,33 +/- 0,598 | 0,373 +/- 0,121 | 0,202 +/- 0,211 | 5,87 +/- 0,171 | 9,83 +/- 5,49 |
| **CAT** | 0,528 +/- 0,0642 | 0,0659 +/- 0,00408 | 0,115 +/- 0,149 | 0,349 +/- 0,0754 | 0,575 +/- 0,0704 |
| **APX** | N.D. | 0,765 +/- 0,4 | 0,547 +/- 0,631 | N.D. | 1,47 +/- 0,642 |
| **MDHAR** | 0,686 +/- 0,154 | N.D. | N.D. | 0,744 +/- 0,0646 | 0,228 +/- 0,0369 |
| **DHAR** | 1,22 +/- 0,124 | N.D. | N.D. | 0,566 +/- 0,166 | 0,179 +/- 0,0235 |
| **GR** | 1,62 +/- 0,0402 | 0,463 +/- 0,243 | 0,0543 +/- 0,0841 | N.D. | 0,377 +/- 0,159 |
| **GST** | 1,97 +/- 0,137 | 0,00505 +/- 0,0022 | 0,0209 +/- 0,000889 | 6,86 +/- 0,196 | 3,89 +/- 0,251 |

Table S2: **Activity profile of the nine antioxidant enzymatic signature in different plants and tissue**. The values are normalized per mg protein (Mean v [nkat/mg prot]) ± standard deviation; N.D. not detectable. The activities for Arabidopsis leaves, maize leaves and maize roots are average of 3 independent plant replicates whereas for sugar beet roots and barley leaves 3 distinct extractions were performed on the same plant sample, consisting of at least 15 individual plants for barley leaves and X for sugar beet roots.
