# Supplementary material for: Simple semi-high throughput determination of activity signatures of key antioxidant enzymes for physiological phenotyping
Source: Plant Methods. 2020 Mar 21;16:42. doi: 10.1186/s13007-020-00583-8 (PMC7085164; doi:10.1186/s13007-020-00583-8)
Supplement: Supplementary file 3 — Additional file 3: Figure S1. Antioxidant enzymatic activities during drought. Nicotiana tabacum plants were grown for 60 days in the greenhouse, thereafter, water was withheld for half of the plants whereas the other half was kept watered. Leaves were harvested every two days for a total of 14 days after watering was stopped and the enzymatic activities of the 9 antioxidant scavenging enzymes were tested and normalized by protein content. Bars indicate standard deviations of three independent biological replicates. [file 13007_2020_583_MOESM3_ESM.docx]

Figure S1: **Antioxidant enzymatic activities during drought**. *Nicotiana tabacum* plants were grown for 60 days in the greenhouse, thereafter, water was withheld for half of the plants whereas the other half was kept watered. Leaves were harvested every two days for a total of 14 days after watering was stopped and the enzymatic activities of the 9 antioxidant scavenging enzymes were tested and normalized by protein content. Bars indicate standard deviations of three independent biological replicates.
