# Supplementary material for: Simple semi-high throughput determination of activity signatures of key antioxidant enzymes for physiological phenotyping
Source: Plant Methods. 2020 Mar 21;16:42. doi: 10.1186/s13007-020-00583-8 (PMC7085164; doi:10.1186/s13007-020-00583-8)
Supplement: Supplementary file 4 — Additional file 4: Figure S2. Antioxidant enzymatic activities during salt stress. Nicotiana tabacum plants were grown for 60 days in the greenhouse, thereafter, detached leaves were fed with 0,2M NaCl or 0,5M NaCl via their petioles. Leaves were sampled every two days for a total of 14 days after NaCl application and the enzymatic activities of the 9 antioxidant scavenging enzymes were tested and normalized by protein content. Bars indicate standard deviations of three independent biological replicates. [file 13007_2020_583_MOESM4_ESM.docx]

Figure S2: **Antioxidant enzymatic activities during salt stress**. *Nicotiana tabacum* plants were grown for 60 days in the greenhouse, thereafter, detached leaves were fed with 0,2M NaCl or 0,5M NaCl via their petioles. Leaves were sampled every two days for a total of 14 days after NaCl application and the enzymatic activities of the 9 antioxidant scavenging enzymes were tested and normalized by protein content. Bars indicate standard deviations of three independent biological replicates.
