# Supplementary material for: Simple semi-high throughput determination of activity signatures of key antioxidant enzymes for physiological phenotyping
Source: Plant Methods. 2020 Mar 21;16:42. doi: 10.1186/s13007-020-00583-8 (PMC7085164; doi:10.1186/s13007-020-00583-8)
Supplement: Supplementary file 5 — Additional file 5: Figure S3. Antioxidant enzymatic activities during pathogen stress. Nicotiana tabacum plants were grown for 60 days in the greenhouse, thereafter, the plants were treated with either the plant pathogen Pseudomonas syringae pv tabaci (Pst) which is virulent in Nicotiana tabacum, the avirulent strain Pseudomonas syringae pv phaseolicola (Psp), mechanical wounding, MgCl2 as a control or untreated plants. Leaves were harvested at 2, 8, 24 and 48 hours after treatment and the enzymatic activities of the ten antioxidant scavenging enzymes were tested and normalized by protein content. Bars indicate standard deviations of three independent biological replicates. [file 13007_2020_583_MOESM5_ESM.docx]

Figure S3: **Antioxidant enzymatic activities during pathogen stress**. *Nicotiana tabacum* plants were grown for 60 days in the greenhouse, thereafter, the plants were treated with either the plant pathogen *Pseudomonas syringae* pv *tabaci* (Pst) which is virulent in *Nicotiana tabacum*, the avirulent strain *Pseudomonas syringae* pv *phaseolicola* (Psp), mechanical wounding, MgCl_2_ as a control or untreated plants. Leaves were harvested at 2, 8, 24 and 48 hours after treatment and the enzymatic activities of the ten antioxidant scavenging enzymes were tested and normalized by protein content. Bars indicate standard deviations of three independent biological replicates.
